# Mass Spectrometric Study of the Most Common Potential Migrants Extractible from the Inner Coatings of Metallic Beverage Cans

**DOI:** 10.3390/foods13132025

**Published:** 2024-06-26

**Authors:** Monika Beszterda-Buszczak, Małgorzata Kasperkowiak, Artur Teżyk, Natalia Augustynowicz, Rafał Frański

**Affiliations:** 1 Department of Food Biochemistry and Analysis, Poznań University of Life Sciences, Mazowiecka 48, 60-623 Poznań, Poland; monika.beszterda@gmail.com; 2 Centre for Advanced Technologies, Adam Mickiewicz University, Uniwersytetu Poznańskiego 10, 61-614 Poznań, Poland; malgorzata.kasperkowiak@amu.edu.pl; 3 Department of Forensic Medicine, Poznań University of Medical Sciences, Rokietnicka 10, 60-806 Poznań, Poland; atezyk@ump.edu.pl; 4 Faculty of Chemistry, Adam Mickiewicz University, Uniwersytetu Poznańskiego 8, 61-614 Poznań, Poland; nataug4@st.amu.edu.pl

**Keywords:** canned sweetened beverages, can coating material, bisphenol A diglycidyl ether, butoxyethanol, HPLC-MS, fragmentation pathways

## Abstract

Population exposure to endocrine disrupting chemical- bisphenols, which are used commonly in food containers and drinking water pipes in Europe, is above acceptable health and safety levels, according to updated research data. In order to evaluate the most abundant potential migrants in canned sweetened beverages marketed in Poland, we performed the HPLC-MS screening test of the migrants present in the can coating material. The analyzed samples represented the three top-ranked companies of the global soft drink market; it is reasonable to assume that the obtained data are of global validity. The tested can coatings and beverages contained bisphenols conjugates such as five butoxyethanol (BuOEtOH) adducts with bisphenol A diglycidyl ether (BADGE), one butoxyethanol adduct with bisphenol A monoglycidyl ether (BAMGE), and cyclo-di-BADGE. The performed HPLC-MS/MS analysis in the MRM mode enabled evaluation of the concentrations of the detected conjugates in canned beverages which were found to be very low, namely at the level of 1 µg/L. On the other hand, the high consumption of canned beverages may yield a risk associated with the presence of these compounds in the diet. The subsequent HPLC-QTOF-MS/MS experiments allowed, for the first time, a detailed determination of the fragmentation pathways of the detected migrants as well as detection of the isomers of the two migrants, namely BADGE + BuOEtOH and BADGE + BuOEtOH + HCl.

## 1. Introduction

Excessive consumption of sweetened beverages is usually associated with the problem of obesity, cardiometabolic diseases or with the risk of diabetes [1,2]. On the other hand, since vast amounts of sweetened beverages are sold in canned form, there is also a risk associated with the presence of migrants from can coating materials, mainly bisphenol A (BPA), bisphenol A diglycidyl ether (BADGE) and their derivatives. Studies on the presence of these compounds in canned beverages are rather limited in comparison to those related to the presence of these compounds in other canned foodstuffs, and the concentrations of these compounds in beverages are very low (usually <1 µg/L), in comparison to those in the other canned foodstuffs [3]. Therefore, the consumption of canned foodstuffs may be associated with the higher levels of urinary BPA concentrations; however, the consumption of canned beverages has not been associated with them [4]. In a number of studies, BPA has been detected in the analyzed beverages [5,6,7,8,9,10,11,12,13], although there is also a recent report that indicates the absence of BPA in canned beverages [14]. Reports of the detection of BADGE and its derivatives in canned beverages are not numerous. For example, BADGE + 2H_2_O has been detected in canned soft drink beverages, e.g., cola, soda, tea [15,16]. Gallart-Ayala et al. detected BADGE and BADGE + 2H_2_O as the most abundant migrants in such beverages as citrus soda, apple soda, lemon beer [17]. Usually, the concentrations of BADGE and its derivatives were at the level of 10 µg/L. Recently, Xie et al. detected BADGE and its derivatives in canned grape juice and the most abundant was BADGE + 2HCl, but its concentration was very low (about 0.6 µg/L) [18]. It has also been found that BADGE is quite abundant and a quite common migrant in canned beer, whereas it is a very rare migrant in canned energy drinks [19,20]. There are also reports in which BADGE and its derivatives have not been detected in canned beverages, which of course may be related to the limit of detection of the used analytical method [21,22].

Although the concentrations of BPA, BADGE and their derivatives in canned beverages are usually very low, the high consumption of canned beverages may yield a risk associated with the presence of these compounds [23,24]. In the EU canned beverages account for about 60% of the consumption of total canned foodstuffs [25]. Average daily consumption of canned beverages in China is much higher than daily consumption of other canned foodstuffs [12]. The number of beverage cans sold per annum in 2005 in the UK was 6378.6 × 10^6^, whereas that of food cans was 4960.2 × 10^6^ [26]. In some countries sweetened beverages are probably consumed by children daily [1,2,10], and it is plausible that most of them are canned beverages.

In order to evaluate the most common and the most abundant migrants in canned sweetened beverages marketed in Poland, we performed the HPLC-MS screening test of the migrants present in the can coating material. We tested twelve cans (canned beverages were purchased from local markets in Western Poland) which represented the three top-ranked companies on the global soft drink market [27]. The number of analyzed samples may seem rather small; however, the selection of common canned sweetened beverages available in Poland is not high and our choice covers those which are consumed the most frequently. The experiment was repeated after 1 year on cans obtained from different markets. The detected migrants were further analyzed in detail by quadrupole time-of-flight mass spectrometry (HPLC-QTOF-MS/MS).

## 2. Materials and Methods

### 2.1. Sample Preparation

All carbonated drinks with volume per piece of 0.33 L were purchased from local markets in Western Poland. Since acetonitrile is one of the most common solvents widely used for the extraction of migrants from can coatings [22,28,29,30,31], the empty cans (not rinsed with water or other solvents), were filled with 50 mL of acetonitrile (Super Purity Solvent, Romil Ltd., Cambridge, UK), and the extraction process was carried out by vigorous stirring of the solution for 90 min. In our experiment, cans filled with acetonitrile were placed into the 6-place stirring block (HS 260 Control, IKA, Staufen im Breisgau, Germany) and their contents were mixed in a reciprocating motion for 90 min at medium speed (as fast as possible for open cans). The extracts were concentrated to a minimum by vacuum evaporation (Rotavapor R-210, Büchi, Flawil, Switzerland) and made up to a final volume of 3 mL with acetonitrile. Before transfer to vials, the samples were further filtered through syringe filters with a pore size of 0.45 μm (Macherey-Nagel GmbH, Düren, Germany).

For analysis of the content of the cans, a 50 mL sample of each beverage was degassed by sonication (Ultrasonic washer Sonic-3, Polsonic, Poznań, Poland) for 20 min. Then, 5 mL was loaded into an SPE cartridge (Oasis Prime HLB, 60 mg, Waters^®^, Milford, MA, USA) that was previously conditioned with 3 mL of methanol and 3 mL of deionized water. The water used was deionized by using the Spring 20 demineralizer (Hydrolab, Straszyn, Poland). The conductivity of the purified water was <0.1 μS/cm. Finally, the analytes were eluted with 4 mL of methanol (Super Purity Solvent, Romil Ltd., Cambridge, UK). The collected fraction was evaporated to dryness and the extract reconstituted with 1 mL of acetonitrile (Super Purity Solvent, Romil Ltd., Cambridge, UK).

### 2.2. HPLC-MS Screening Test and MRM Analysis

**Figure 1:**
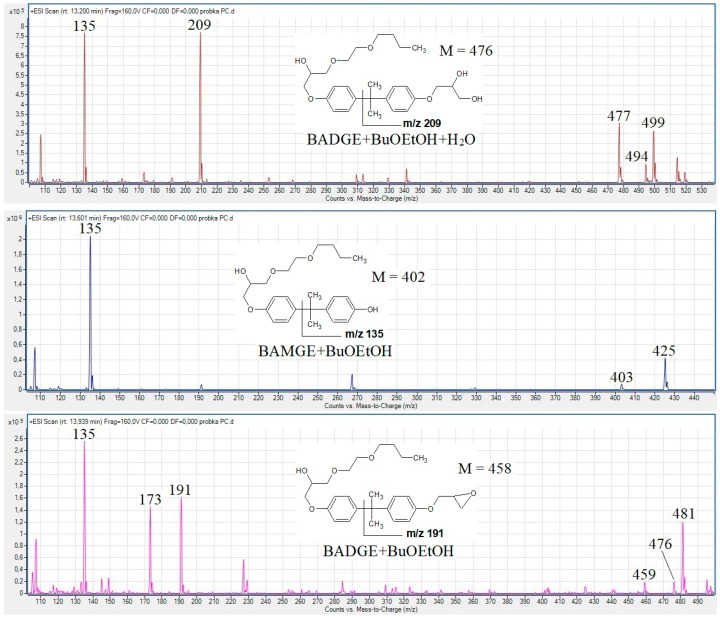

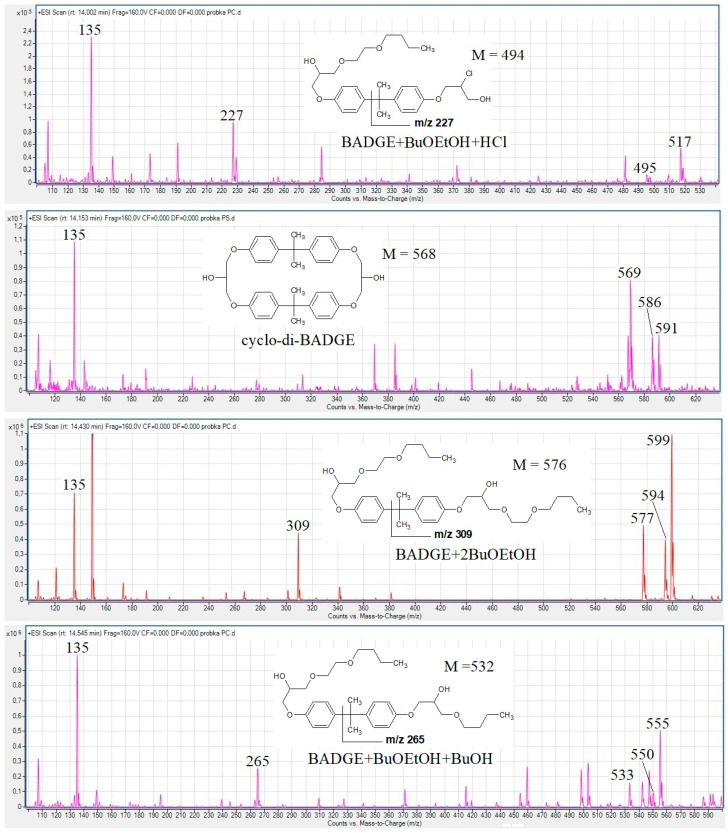
The obtained ESI mass spectra, compound structures (in order of elution) and formation of the characteristic diagnostic product ions.

The samples were analyzed by high-pressure liquid chromatography (Agilent 1200 series, Waldbronn, Germany) coupled with a triple quadrupole mass spectrometer, equipped with an electrospray ionization source (Agilent 6410B, Wildnington, DE, USA). The mass spectrometer was working in the full scan positive ion mode. The column used was a Poroshell 120 EC C18, 75 × 3.0 mm i.d., 2.7 µm particle size, with a dedicated precolumn 5 mm × 3.0 mm i.d, 2.7 µm particle size (Agilent, USA). The mobile phases were: 0.1% formic acid in water (solvent A, pH ≈ 3) and 0.1% formic acid in acetonitrile (solvent B) (Merck, Darmstad, Germany). The flow rate was 0.5 mL/min. The gradient was programmed as follows: 95% A and 5% B for 3 min, followed by a linear change to 20% A and 80% B in 8 min and to 5% A and 95% B in 13 min, held for 7 min. Source parameters were as follows: drying gas (nitrogen) temperature 350 °C, gas flow 10 L/min, nebulizing gas pressure 40 psi, capillary voltage 4000 V, fragmentor voltage was set at 160 V. This parameter has the greatest impact on the full scan mass spectra recorded. An increase in this parameter leads to the so-called “in-source” fragmentation/dissociation, but a too-low fragmentor voltage may cause a decrease in sensitivity. The used voltage made it possible to obtain the mass spectra containing peaks of precursors ions ([M + H]^+^, [M + NH_4_]^+^, [M + Na]^+^), peaks of diagnostic ions and abundant product ion at *m*/*z* 135–HO-C_6_H_4_-C^+^(CH_3_)_2_ (Figure 1).

In order to detect the analyzed adducts in the beverages samples, the analyses were performed in the multi-reaction monitoring mode (*m*/*z* of [M + H]^+^ → *m*/*z* 135). For each compound, the fragmentor voltage and collision energy were adjusted to reach the highest sensitivity (50–200 V, 10–40 eV).

### 2.3. HPLC-QTOF-MS Analysis

The HPLC-QTOF-MS analyses were performed using an UltiMateTM 3000 UHPLC system (ThermoScientific/Dionex, Sunnyvale, CA, USA) and an Impact HD mass spectrometer (QTOF type instrument equipped with electrospray ion source; Bruker Daltonics, Billerica, MA, USA). The instrument was previously calibrated with the standard tune mixture. Using an autosampler, the sample solutions were injected onto the Kinetex C18 column (100 × 2.10 mm i.d., 2.6 μm particle size). The used mobile phases were water with 0.1% of formic acid (solvent A) and acetonitrile with 0.1% of formic acid (solvent B). The samples were analyzed using a linear gradient with a flow rate of 0.3 mL/min and the column temperature was maintained at 35 °C. The gradient was programmed as follows: 90% A and 10% B for 1 min, followed by a linear change to 5% A and 95% B in 20 min, held for 10 min. The instrument was operated in the positive ion mode, under the following optimized settings: end-plate voltage 500 V, capillary voltage 4.2 kV; collision energy 8 eV, nebulizer pressure 1.5 bar; dry gas (nitrogen); temperature 200 °C; dry gas flow rate 8 L/min. In order to obtain the product ion spectra (HPLC-QTOF-MS/MS analysis), the collision energy was increased to 35–37 eV.

## 3. Results and Discussion

### 3.1. Detection of BADGE-BuOEtOH Adducts, BAMGE + BuOEtOH and Cyclo-di-BADGE as the Most Common Potential Migrants

In all twelve tested can coatings and the corresponding beverages, we detected five butoxyethanol adducts with BADGE (BADGE + BuOEtOH, BADGE + 2BuOEtOH, BADGE + BuOEtOH + BuOH, BADGE + BuOEtOH + H_2_O, BADGE + BuOEtOH + HCl), one butoxyethanol adduct with bisphenol A monoglycidyl ether (BAMGE + BuOEtOH) and cyclo-di-BADGE as the most abundant BADGE conjugates. The obtained ESI mass spectra, compound structures and formation of characteristic diagnostic product ions are shown in Figure 1, and extracted ion chromatograms are shown in the supplementary material. The diagnostic product ions have the general formula (CH_3_)_2_C^+^-C_6_H_4_-OR, and their *m*/*z* allow determination of R which is of importance with respect to the structure determination of the unknowns. The ions accurate masses obtained from the high-resolution mass spectra (HPLC-QTOF-MS analyses) confirmed their elemental compositions, thus the structures of the detected adducts, as shown in Table 1 for [M + H]^+^ ions.

It has to be stressed that BADGE and BPA were not detected in the analyzed extract of the can coating materials (in order to detect BPA the analyses were performed in the negative ion mode), similarly to a few other works [19,20,21,22], which of course may be related to the concentrations of these compounds being below the detection limit.

Cyclo-di-BADGE, formally BPA-BADGE adduct, is one of the most common migrants [30,32,33], whereas BuOEtOH-BADGE adducts are definitely less numerous. Schaefer and Simat described the synthesis of a number of BuOEtOH-BADGE adducts and their analysis focuses on the extracts obtained from single-side coated tinplate strips, coated with different types of epoxy coatings [34]. The strips were provided by the Valspar Corporation. Similarly, Bradley et al. identified a number of BuOEtOH-BADGE adducts in the extracts of epoxy phenolic-coated tin plates which were also provided by Valspar [29]. Paseiro-Cerrato et al. identified different kinds of BuOEtOH-BADGE adducts in the extracts of epoxy coatings provided by their industrial partner [31]. Berger and Oehme (2000) identified the BuOEtOH-BADGE adducts in the extracts of a BADGE-based can which was obtained directly from a food can factory in Switzerland [28]. BuOEtOH-BADGE adducts have also been detected in a few extracts of some food can coatings [35,36]. Lestido-Cardama et al. have tentatively identified BuOEtOH-BADGE adducts in one beverage can coating [37]. Recently, Bustos et al. found that the adduct BADGE + H_2_O + BuOEtOH is one of the most common migrants present in canned foods [32].

**Figure 2:**
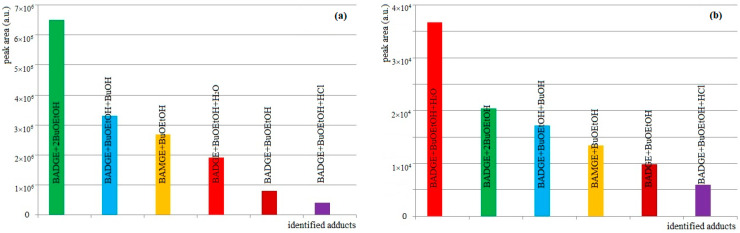
Relative abundances of the detected adducts in the extracts of can coating materials (**a**), in the beverage sample (**b**).

Our results, and those of Lestido-Cardama et al. [37], indicate that BuOEtOH-BADGE adducts are nowadays the most common and most abundant migrants present in beverage can coating materials (the fact that these adducts are less numerous in the can coating materials of other foodstuffs may be associated with the various types of BADGE-based epoxy coatings for cans [38]), which should initiate the targeted precision analysis of canned beverages. However, such an analysis would require purity standards which are not available. To the best of our knowledge, the synthesis of BuOEtOH-BADGE adducts described by Schaefer and Simat has not yet been repeated [34]. Therefore, it has not been possible to carry out methodological assessments such as sample recovery, linear range, detection limit and quantification limit. On the other hand, we decided to use BADGE as a standard to estimate the concentrations of the detected BuOEtOH-BADGE adducts, assuming that BADGE and its adducts have comparable ESI-MS responses. Their concentrations were estimated by comparing the extracted ion chromatogram peak areas and, in the extracts of can coating materials, the estimated concentrations were on the level 1–10 mg/L in the following order: BADGE + 2BuOEtOH > BADGE + BuOEtOH + BuOH > BAMGE + BuOEtOH > BADGE + BuOEtOH + H_2_O > BADGE + BuOEtOH > BADGE + BuOEtOH + HCl (Figure 2a). In the beverage samples, the BuOEtOH-BADGE adducts were also detected but only during the targeted analysis by using multi-reaction monitoring modes (*m*/*z* of [M + H]^+^ → *m*/*z* 135). The estimated concentrations were on the level of 1 µg/L in the following order: BADGE + BuOEtOH + H_2_O > BADGE + 2BuOEtOH > BAMGE + BuOEtOH > BADGE + BuOEtOH + BuOH > BADGE + BuOEtOH > BADGE + BuOEtOH + HCl (Figure 2b). It is clear that the presence of hydroxy groups increases the water solubility of the adduct BADGE + BuOEtOH + H_2_O, which increases its migration from the can coating materials to the canned beverages.

### 3.2. The Fragmentation Pathways of the Detected Migrants as Studied by HPLC-QTOF-MS/MS Analysis

**Figure 3:**
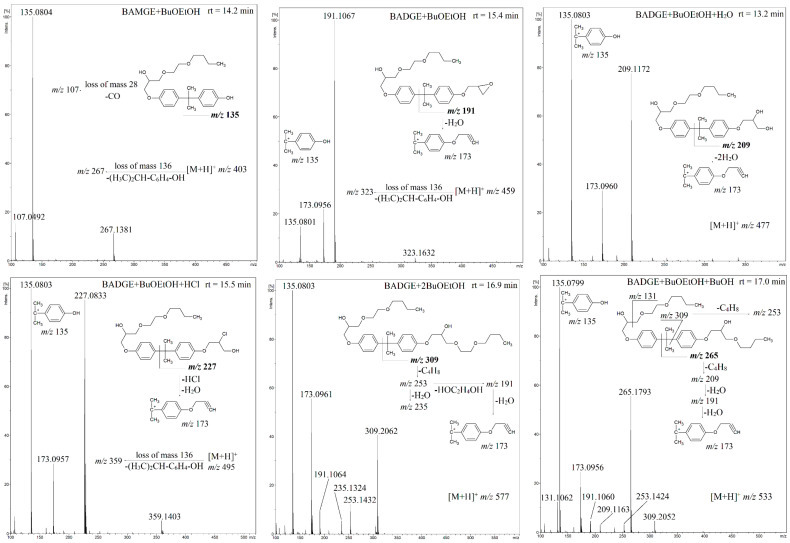
Product ion spectra of [M + H]^+^ ions of BADGE-BuOEtOH adducts and BAMGE + BuOEtOH.

In all twelve tested can-coatings and the corresponding beverages, we detected five butoxyethanol adducts with BADGE (BADGE + BuOEtOH, BADGE + 2BuOEtOH, BADGE + BuOEtOH + BuOH, BADGE + BuOEtOH + H_2_O, BADGE + BuOEtOH + HCl), one butoxyethanol adduct with bisphenol A monoglycidyl ether (BAMGE + BuOEtOH) and cyclo-di-BADGE as the most abundant BADGE conjugates. Figure 2 shows the product ion spectra of the [M + H]^+^ ions of BADGE-BuOEtOH adducts and BAMGE + BuOEtOH obtained at collision energies of 35 eV (at this collision energy, the parent ions are not detected) and the observed fragmentation pathways. For each of the compounds studied, besides BADGE + BuOEtOH, the most abundant (100% ri) was the product ion at *m*/*z* 135 (for BADGE-BuOEtOH, this ion had 15% ri). For BAMGE + BuOEtOH, there was also a product ion at *m/z* 267 (loss of mass 136) which can be regarded as a diagnostic ion of BAMGE conjugates (Figure 3).

On the other hand, the loss of mass of 136 was also observed for BADGE + BuOEtOH and BADGE + BuOEtOH + HCl (the product ions at *m*/*z* 323 and 359 were not abundant but clearly seen, and it is difficult to propose even their tentative structures, Figure 3), which can be regarded as an interesting example of the loss of the internal part of the fragmented ion [39]. Thus, the loss of mass of 136 is not limited to the BAMGE conjugates and may confuse the structure determination. Product ions at *m*/*z* 173 were observed for all compounds besides BAMGE + BuOEtOH (Figure 3). Therefore, the lack of this ion can be regarded as a diagnostic feature of BAMGE conjugates.

It has to be pointed out that the measured accurate *m*/*z* values of the product ions fully confirm their elemental composition, thus the proposed fragmentation pathways (e.g., the measured accurate *m*/*z* 309.2062 (Figure 3) match perfectly the exact *m/z* of [C_18_H_29_O_3_]^+^ which is 309.2061).

Although cyclo-di-BADGE is one of the most common migrants [30,32,33], its fragmentation pathways have not yet been studied in detail. The product ion spectrum of cyclo-di-BADGE and the observed fragmentation pathway (and a brief discussion) are shown in the Supplementary Materials (Figure S2).

### 3.3. Migrant Isomers Detected upon HPLC-QTOF-MS/MS Analysis

**Figure 4:**
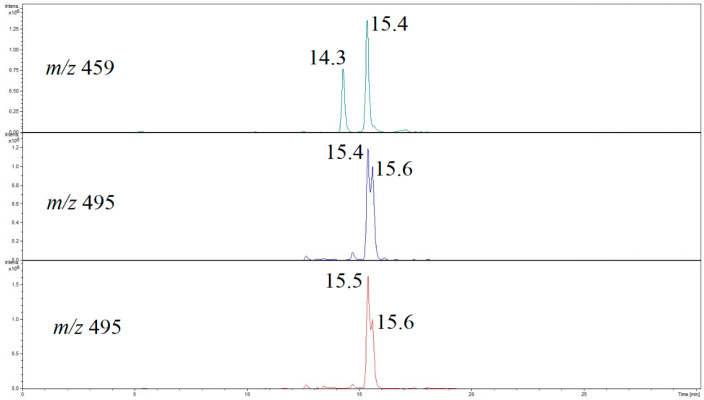
Extracted ion chromatogram of the [M + H]^+^ ions of BADGE + BuEtOH isomers (*m*/*z* 459) and BADGE + BuEtOH + HCl isomers (*m*/*z* 495).

**Figure 5:**
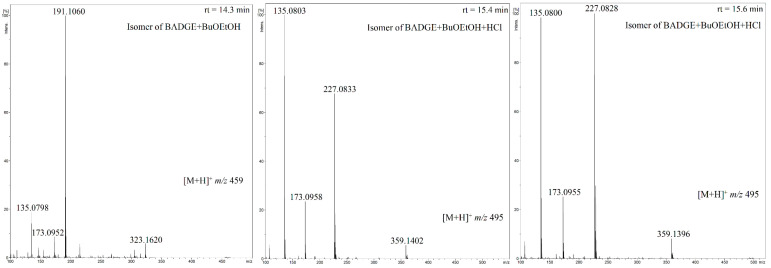
Product ion spectra of the [M + H]^+^ ions of BADGE + BuEtOH isomer and BADGE + BuEtOH + HCl isomers.

Although the LC conditions used for HPLC-QTOF-MS analysis have not always enabled the full chromatographic separation of the detected migrants (e.g., BADGE + BuOEtOH rt = 15.4 min and BADGE + BuOEtOH + HCl rt = 15.5 min, BADGE + 2BuOEtOH rt = 16.9 min and BADGE + BuOEtOH + BuOH rt = 17.0 min, Figure 3), they enabled separation (detection) of two isomers of BADGE + BuOEtOH (rt = 14.3 and 15.14 min) and three isomers of BADGE + BuOEtOH + HCl (rt = 15.4, 15.15 and 15.16 min). Figure 4 and Figure 5 show the exemplary extracted ion chromatograms and product ion spectra, respectively.

**Figure 6:**
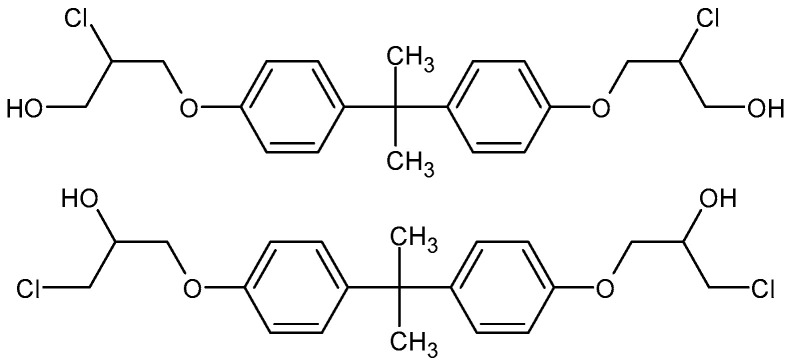
Two different structures of BADGE + 2HCl related to the different HCl addition patterns to the epoxide ring.

To the best of our knowledge, the detection of BADGE migrant isomers has not yet been claimed. By comparison with the isomers mass spectra (Figure 3 and Figure 5), it is clear that the fragmentation patterns of the isomers differ only by the relative abundances of product ions. Therefore, it is difficult to propose unambiguously the structures of the isomers. On the other hand, there are reported examples of different structures of BADGE adducts related to different addition patterns of the epoxide ring. For example, two different structures, shown in Figure 6, have been reported for BADGE + 2HCl by Gallart-Ayala et al. and by Xue et al. [15,40]. Therefore, it is reasonable that the detected isomers of BADGE + BuOEtOH and BADGE + BuOEtOH + HCl differ by addition patterns of HCl and/or BuOEtOH to the epoxide ring.

## 4. Conclusions

Five BADGE-BuOEtOH adducts, the BAMGE + BuOEtOH adduct and cyclo-di-BADGE were found to be the most common migrants in canned beverages. The analyzed samples represented the three top-ranked companies of the global soft drink market, thus it is reasonable to assume that the obtained data are of global validity. The concentrations of the analyzed adducts in the beverages were very low, which made it possible to draw the conclusion that only huge and regular consumption of canned beverages may cause the health risk associated with these adducts. On the other hand, BADGE and its derivatives might be endocrine disruptors according to the available toxicology data [41,42,43]; therefore, more detailed studies are still needed. For each of the adducts, a detailed fragmentation pathway of [M + H]^+^ ions has been proposed. The adducts BADGE + BuOEtOH and BADGE + BuOEtOH + HCl have been found to be somewhat intriguing from the analytical point of view; namely, the loss of the internal part of the fragmented ion has been observed (i), the isomers of BADGE + BuOEtOH and BADGE + BuOEtOH + HCl have been detected (ii).

## Figures and Tables

**Table 1:** Confirmation data of elemental composition of [M + H]^+^ ions.

Compound	Composition of [M + H]^+^	Exact Mass	Measured Mass	Error (ppm)
BAMGE + BuOEtOH	C_24_H_35_O_5_	403.2479	403.2470	−2.2
BADGE + BuOEtOH	C_27_H_39_O_6_	459.2742	459.2726	−3.5
BADGE + BuOEtOH + H_2_O	C_27_H_41_O_7_	477.2847	477.2840	−1.6
BADGExBuOEtOH + HCl	C_27_H_40_O_6_Cl	495.2508	495.2499	−1.8
BADGE + BuOEtOH + BuOH	C_31_H_49_O_7_	533.3473	533.3464	−1.7
BADGE + 2BuOEtOH	C_33_H_53_O_8_	577.3735	577.3725	−1.7

## Data Availability

The original contributions presented in the study are included in the article/Supplementary Materials, further inquiries can be directed to the corresponding author.

## Supplementary Materials

The following supporting information can be downloaded at: https://www.mdpi.com/article/10.3390/foods13132025/s1, Figure S1: Extracted ion chromatograms of [M + Na]^+^ ions; Figure S2: Product ion spectrum of [M + H]^+^ ion of cyclo-di-BADGE.
