# Supplementary material for: Mass Spectrometric Study of the Most Common Potential Migrants Extractible from the Inner Coatings of Metallic Beverage Cans
Source: Foods. 2024 Jun 26;13(13):2025. doi: 10.3390/foods13132025 (PMC11241440; doi:10.3390/foods13132025)
Supplement: Supplementary file 1 [file foods-13-02025-s001.zip › foods-3051210-supplementary.pdf]

SUPPLEMENTARY MATERIAL

Mass spectrometric study of the most common potential migrants extractible from inner coatings of metallic beverages cans

Monika Beszterda-Buszczak[a], Małgorzata Kasperkowiak[b], Artur Tezyk[c], Natalia Augustynowicz[d], Rafał Frański[d]

[a] Department of Food Biochemistry and Analysis, Poznań University of Life Sciences, Mazowiecka 48, 60-623 Poznań, Poland

[b] Centre for Advanced Technologies, Adam Mickiewicz University, Uniwersytetu Poznańskiego 10, 61-614 Poznań, Poland

[c] Department of Forensic Medicine, Poznań University of Medical Sciences, Rokietnicka 10, 60-806 Poznań, Poland

[d] Faculty of Chemistry, Adam Mickiewicz University, Uniwersytetu Poznańskiego 8, 61-614 Poznań, Poland

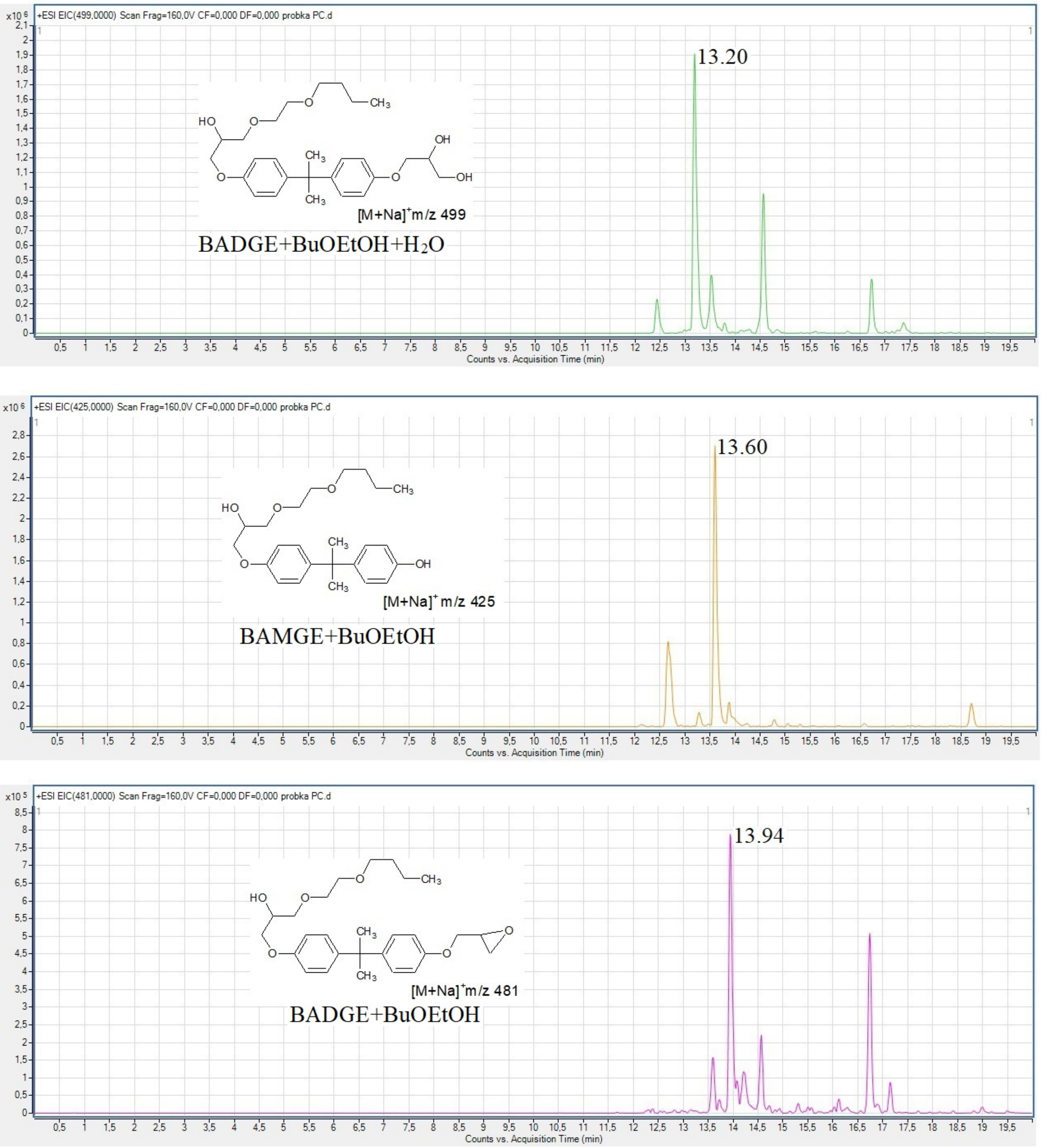

Figure S1. Extracted ion chromatograms of $[M+Na]^+$ ions (peaks assigned by retention times) of the detected migrants.

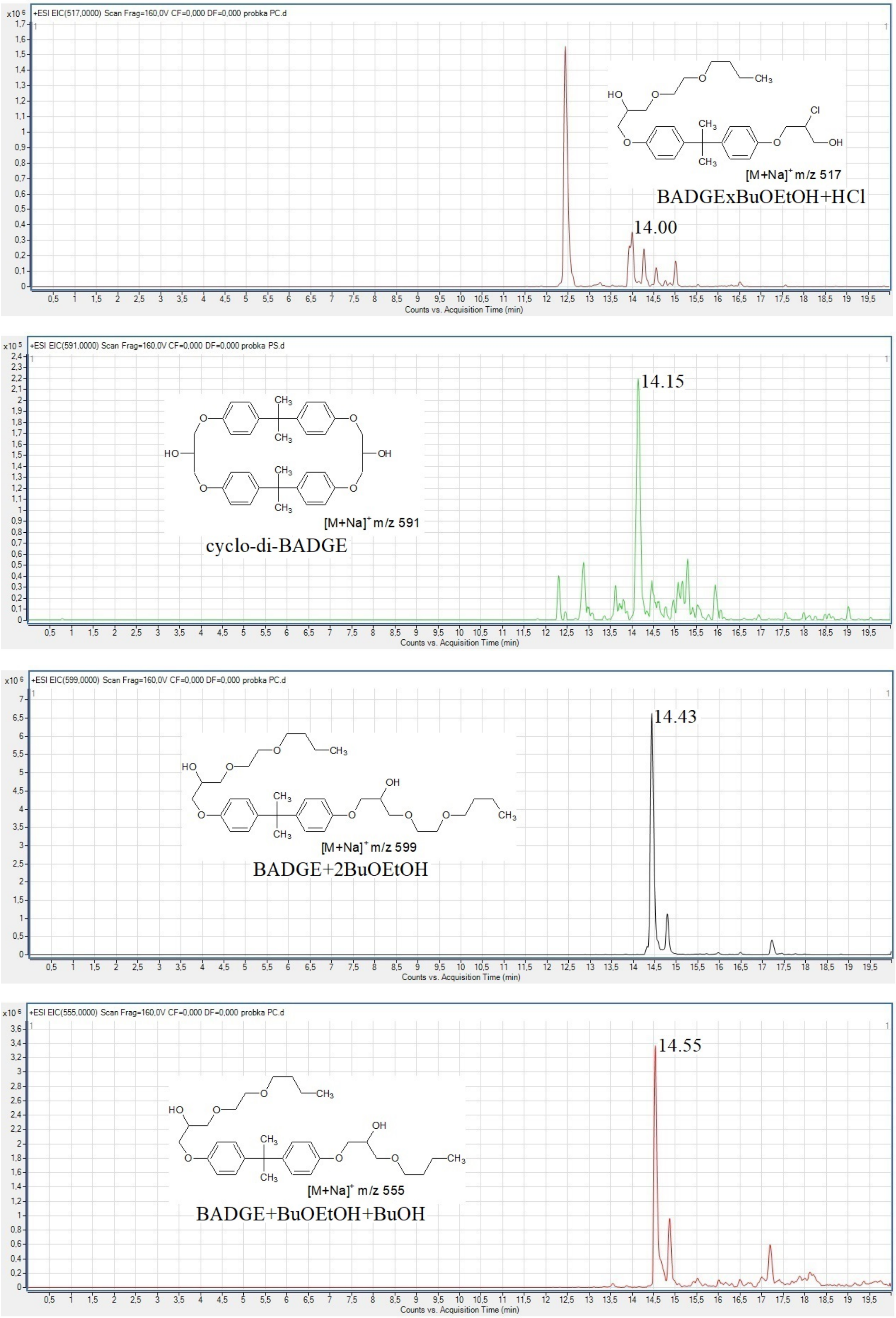

Figure S1. Continuation.

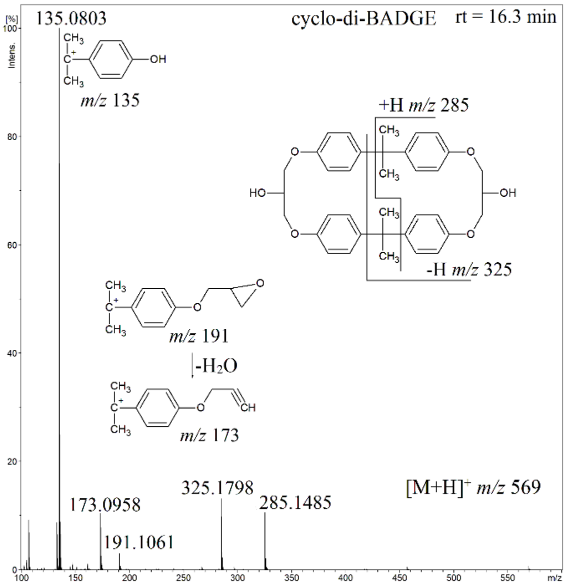

Figure S2. Product ion spectrum of $[M+H]^+$ ion of cyclo-di-BADGE.

The structure of cyclo-di-BADGE is substantially different than those of the others, however, its fragmentation pathway partly resembles those observed for the other migrants (*m/z* 135, 173, 191). The feature which is different from those observed for the other migrants corresponds to the formation of product ions at *m/z* 325 and 285. Since cyclo-di-BADGE is a symmetrical molecule (M=268), the product ion at *m/z* 285 can be regarded as a protonated half of cyclo-di-BADGE. In the above figure one of the possible ways of formation of product ion at *m/z* 285 is shown, and of course the other ways cannot be excluded.
